# Influence of acupuncture twisting parameters on analgesic effects mediated by mast cells in AA rat models

**DOI:** 10.1016/j.bj.2025.100876

**Published:** 2025-06-04

**Authors:** Yi Yu, Yi-Xuan Wang, Xuan Qiao, Ying-Chen Li, Yu-Hang Liu, Zouqin Huang, Wei Yao

**Affiliations:** a College of Medical Instruments, Shanghai University of Medicine & Health Sciences, Shanghai, China; b Shanghai Key Laboratory of Acupuncture Mechanism and Acupoint Function, Department of Aeronautics and Astronautics, Fudan University, Shanghai, China; c Shanghai Pudong New Area Traditional Chinese Medicine Hospital, Shanghai, China

**Keywords:** Acupuncture parameter, Analgesia, Mast cell, AA rat model

## Abstract

Acupuncture has been recognized for its potential effectiveness in pain relief without the side effects commonly associated with pharmaceutical treatments. This study investigates the effects of various acupuncture twisting parameters on analgesia using an acute adjuvant-induced arthritis (AA) rat model, focusing on the roles of mast cell activation, histamine (HIS) release, and local neural pathways. Utilizing robot-assisted acupuncture, we varied rotation angles (60°–360°) and frequencies (0.5–2.5 Hz) to evaluate their influence on pain modulation. The pain threshold recovery ratio (PTRR) was used to quantify the analgesic effect. The optimal combination of a 180° rotation angle and 1.0 Hz frequency resulted in the strongest analgesic effect, as indicated by increased PTRR and elevated mast cell degranulation rates (MCdR). Additionally, local HIS injections replicated the analgesic effects of acupuncture, whereas administration of an H1 receptor antagonist and lidocaine reduced these effects, highlighting the essential roles of HIS and local nerve conduction in acupuncture-induced analgesia. This study demonstrates the existence of optimal acupuncture parameters for achieving maximal analgesic effects in rat models, and elucidates the critical role of mast cell-mediated neural pathways, thus providing insights into optimizing acupunctire's clinical application in pain management.

## Highlights

• Optimal acupuncture parameters (180° angle, 1.0 Hz) improved pain thresholds and mast cell degranulation in AA rats.
• Mast cell degranulation and histamine release played a key role in acupuncture-induced analgesic response.
• Robot-assisted acupuncture enabled precise control of twisting, ensuring repeatability in the experiment.

## Graphical abstract

**Figure:**
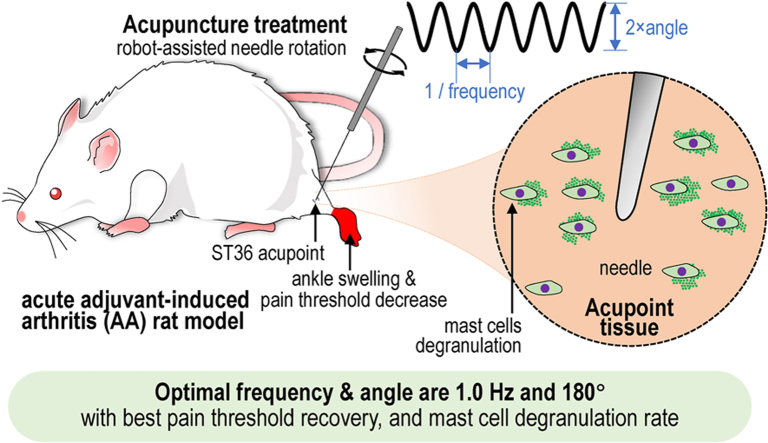
Schematic illustration of acupuncture manipulation on ST36 in an adjuvant arthritis (AA) rat model. Twisting stimulation with 180° angle and 1.0 Hz frequency enhanced mast cell degranulation and improved pain threshold.

## 1 Introduction

As a fundamental component of traditional Chinese medicine, acupuncture involves applying controlled mechanical forces to specific acupoints through precise needle manipulations [1]. These forces alter the local tissue environment, initiating a series of physiological and biochemical responses. Widely recognized for its analgesic effects, acupuncture is frequently used in clinical pain management [2,3]. However, the specific impact of acupuncture parameters, such as duration, frequency, and intensity, on therapeutic outcomes remains partially understood [[4], [5], [6]]. Current acupuncture practices rely heavily on practitioners' individual experience and techniques, often leading to variability in therapeutic effects. This reliance, coupled with insufficient mechanistic studies, has contributed to ongoing debates about acupuncture's clinical efficacy. Clarifying the quantitative influence of specific parameters on therapeutic outcomes and elucidating the mechanisms of acupuncture analgesia are crucial steps toward validating its efficacy and facilitating greater consistency in clinical applications.

Recent clinical and animal studies have progressively uncovered how variations in acupuncture parameters affect therapeutic outcomes, particularly through modifications in twisting angle and frequency, which can significantly alter biological responses and analgesic effects [[7], [8], [9], [10]]. For example, Zhang et al. demonstrated that a twisting-rotating acupuncture session at Neiguan (PC6) in a middle cerebral artery occlusion rat model significantly improved cerebral blood flow and neurological recovery compared to other sessions [11]. Similarly, Song et al. observed that a twisting frequency of 4 Hz produced superior analgesic effects compared to 2 Hz in an acute adjuvant arthritis (AA) rat model [12]. Extending these findings, Min et al. reported that twisting frequency, angle and duration each had significant impacts on acupuncture efficacy, identifying optimal parameters in a kidney yang deficiency rat model as a 90° angle, 180 twists per minute, and a duration of 120 s [13]. Despite these advances, research on acupuncture parameters remains limited, often focusing on isolated variables, with many studies relying on manual operations where parameter control is affected by operator variability. This variability can reduce the stability and precision of parameter application, limiting result consistency and hindering broader application. The robot-assisted system used in this study offers the greater potential for precision and reproducibility in acupuncture practices, addressing these limitations and advancing the standardization of therapeutic protocols.

Research on the biological mechanisms of acupuncture analgesia suggests that its effects are closely related to local immune responses, particularly through the activation of mast cells [14,15]. Studies have shown that acupuncture induces mast cell degranulation, releasing bioactive substances such as histamine (HIS) and serotonin, which play essential roles in anti-inflammatory and analgesic processes [[16], [17], [18], [19], [20], [21]]. Histamine, by binding to local receptors, not only reduces inflammation but can also enhance pain relief by influencing nerve conduction [22,23]. For example, Huang et al. found that acupuncture-induced histamine release significantly alleviated inflammation and enhanced analgesic effects via H1 receptor activation [24]. However, few studies have examined the role of local H1 receptor antagonists, leaving the specific function of histamine in acupuncture mechanisms not fully understood. Additionally, research by Huang et al. demonstrated that local application of lidocaine could weaken acupuncture-induced analgesia, highlighting the critical role of local nerve conduction in pain relief [25]. Therefore, further investigation is warranted into the interactions between nerve conduction and histamine signaling, and their combined effects on acupuncture-induced analgesic mechanisms.

Using a robot-assisted twisting acupuncture system, we varied stimulus parameters, including rotation angle (60°–360°) and frequency (0.5–2.5 Hz), to achieve precise control over the applied stimuli. Pain thresholds were measured at multiple time points pre- and post-treatment to assess therapeutic effects, and mast cell degranulation rates (MCdR) were measured to evaluate cellular responses. Additionally, local histamine injections, H1 receptor antagonists, and lidocaine treatments were applied to further explore the potential biological mechanisms underlying acupuncture-induced analgesia.

## 2 Results

### 2.1 Manual and robot-assisted acupuncture

**Fig. 1:**
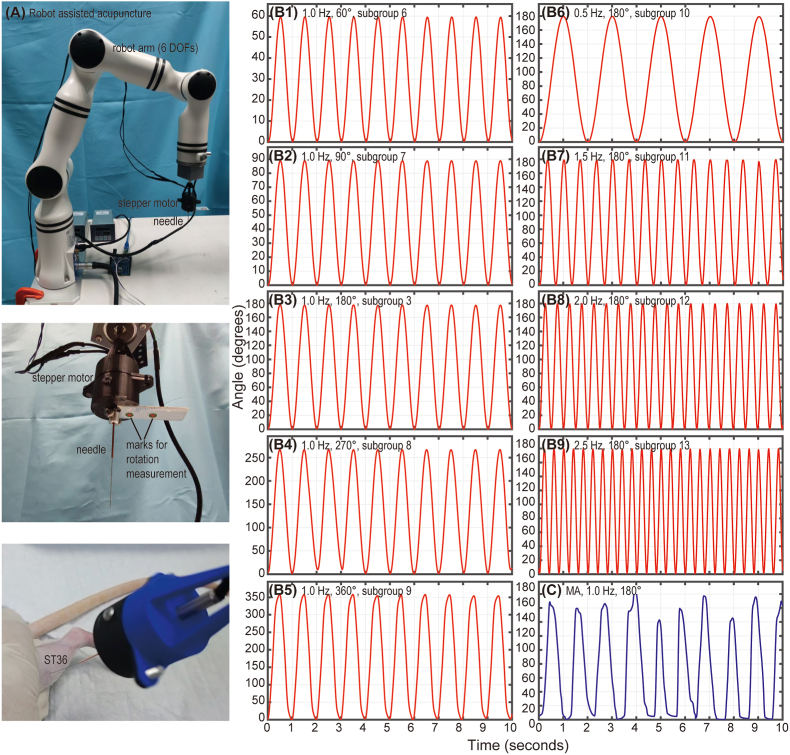
Verification of the robot-assisted acupuncture system. (A) The needle is connected to a stepper motor, which is mounted on a 6-DOF (six degrees of freedom) robot arm. By adjusting the position of the robot arm, the needle can be safely inserted into the ST36 acupoint (left hind limb) of the animal. The stepper motor performed rotational movements to simulate twisting acupuncture technique, with the angle and frequency controlled by a custom Python program. The angular motion was captured via image recognition based on the identified location marks attached to the motor. (B1–B9) demonstrate the angular motion of the needle. The actual movement of the robot-assisted needle manipulation agreed well with our designed parameters (rotation angle and frequency). (C) Representative needle movement during manual acupuncture (MA), which was measured using similar image recognition techniques.

The efficacy of robot-assisted acupuncture (RA) was evaluated by comparing it with manual acupuncture (MA) under similar stimulus parameters: a 5-min duration, a 180° rotation angle, and a 1.0 Hz frequency, as used in a specific RA subgroup. [Fig. 1] presents the verification of the robot-assisted acupuncture system. [Fig. 1A] shows the setup where the needle is connected to a stepper motor, which is mounted on a 6-DOF (six degrees of freedom) robot arm. By adjusting the position of the robot arm, the needle can be safely inserted into the ST36 acupoint (on the left behind limb) of the animal. The stepper motor performs rotational movements to simulate the twisting acupuncture technique, with the angle and frequency controlled by a custom Python program. [Fig. 1B1-B9] further demonstrates the angular motion of the needle, showing that the actual movement of the robot-assisted needle manipulation closely matches the designed parameters for angle and frequency. [Fig. 1C] presents needle movement during MA, measured using a similar image recognition technique.

**Fig. 2:**
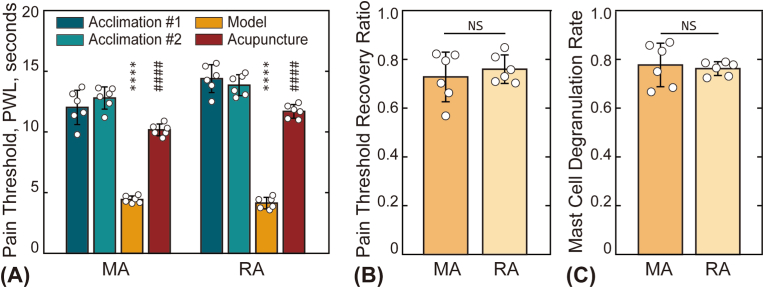
Comparison of robot-assisted acupuncture (RA subgroup) and manual acupuncture (MA group). (A) Pain thresholds of animals in the RA and MA groups at four time points. Asterisks (∗) indicate a statistically significant difference between the baseline pain threshold and post-model establishment values ($L_{0}$ vs. $L_{1}$), ∗∗∗∗*p* < 0.0001. Hash symbols (#) denote a statistically significant difference between pain thresholds before ($L_{1}$) and after ($L_{2}$) treatment, ####*p* < 0.0001. (B) and (C) show the statistical results for the pain threshold recovery ratio (PTRR) and mast cell degranulation rate (MCdR), respectively. No significant difference (NS) was observed between the two groups (*p* > 0.05).

[Fig. 2] compares the results of RA with those of MA. [Fig. 2A] shows the pain thresholds of animals at four time points. In both groups, paw withdrawal latency (PWL) significantly decreased following AA model establishment and partially recovered after treatment, indicating that both manual and robot-assisted acupuncture effectively alleviated pain. A comparison of the pain threshold recovery ratio (PTRR) between the two groups is shown in [Fig. 2B], with values of 0.73 ± 0.10 for the MA group and 0.76 ± 0.06 for the RA subgroup. No statistically significant difference was observed. [Fig. 2C] further demonstrates that the MCdR was also comparable between the groups (0.78 ± 0.09 for MA and 0.76 ± 0.03 for RA).

### 2.2 Effects of different acupuncture parameters on pain threshold

**Fig. 3:**
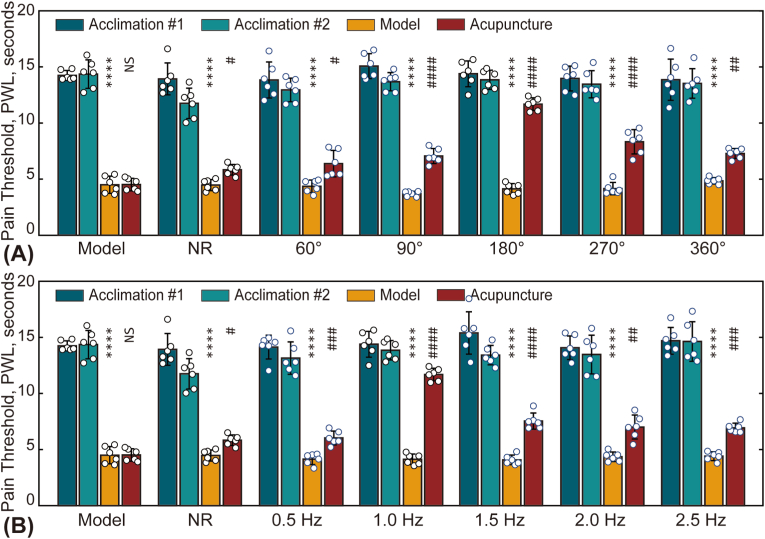
Influence of acupuncture parameters on analgesic effects. (A) Effect of acupuncture rotation angle on pain thresholds. (B) Effect of acupuncture rotation frequency on pain thresholds. In subplots A and B, differences between $L_{1}$ and $L_{0}$, as well as between $L_{2}$ and $L_{1}$, were analyzed. NS: not statistically significant (*p* > 0.05); ∗ or #: *p* < 0.05; ∗∗ or ##: *p* < 0.01; ∗∗∗ or ###: *p* < 0.001; ∗∗∗∗ or ####: *p* < 0.0001.

The effects of acupuncture parameters, specifically stimulus rotation angle and frequency, on analgesic outcomes are illustrated in [Fig. 3]. The impact of rotation angle is shown in [Fig. 3A], comparing five angles (60°, 90°, 180°, 270°, and 360°). The post-treatment pain threshold ($L_{2}$) was angle-dependent, with optimal recovery observed at approximately 180°.

Similarly, the influence of rotation frequency on analgesic outcomes was assessed by comparing five values (0.5, 1.0, 1.5, 2.0, and 2.5 Hz), as shown in [Fig. 3B]. Post-treatment pain threshold ($L_{2}$) varied with frequency, with the most effective recovery occurring at around 1.0 Hz.

### 2.3 Influence of mechanical stimulation on analgesia and mast cell degranulation

**Fig. 4:**
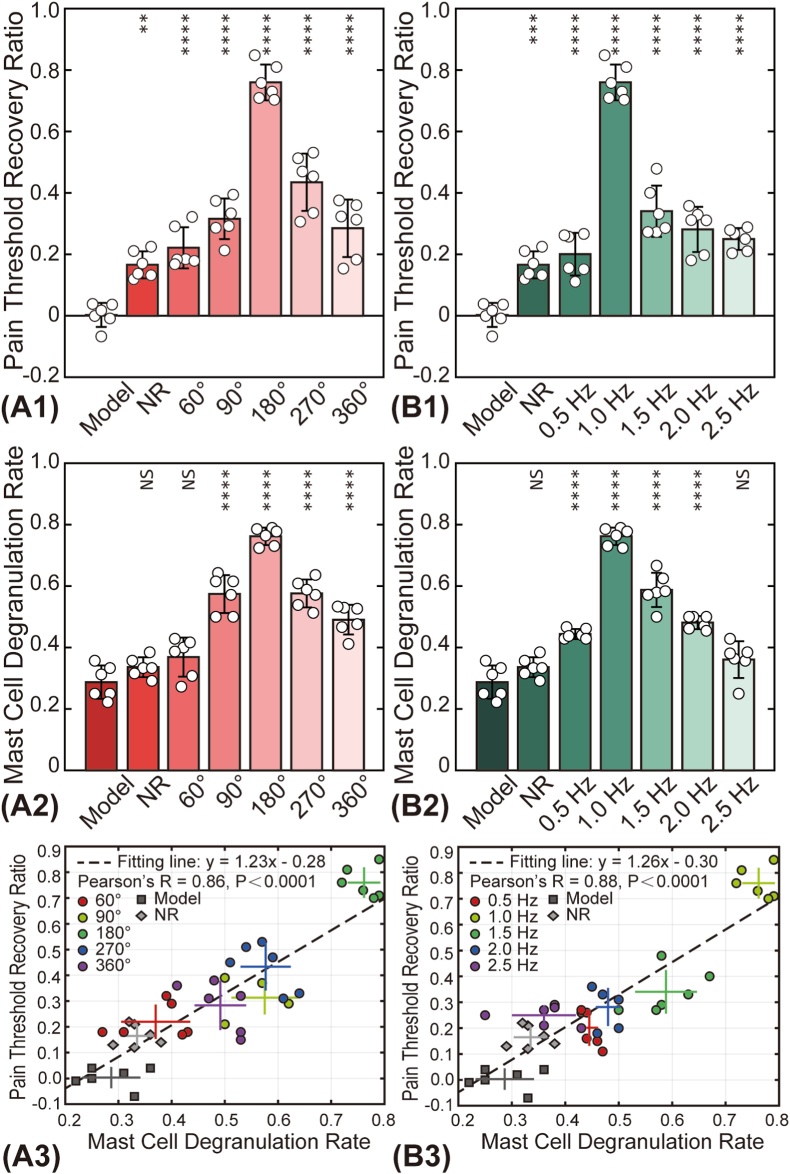
Influence of acupuncture parameters on PTRR and MCdR. (A1-B1) Effect of rotation angle and frequency on PTRR, respectively. (A2-B2) Effect of rotation angle and frequency on MCdR, respectively. The PTRR and MCdR values of other groups were compared pairwise with those of the Model group. NS: not statistically significant (*p* > 0.05); ∗: *p* < 0.05; ∗∗: *p* < 0.01; ∗∗∗: *p* < 0.001; ∗∗∗∗: *p* < 0.0001. (A3-B3) Correlation analysis between PTRR and MCdR. Each dot represents one animal, and colors denote different groups. Cross symbols indicate the mean ± standard deviation for each group.

[Fig. 4] illustrates the effects of various acupuncture parameters on analgesic efficacy and MCdR at the ST36 acupoint. The PTRR values [Fig. 4A1, 4B1] indicate that the optimal analgesic effect was achieved with a combination of 5-min duration, a 180° rotation angle, and a 1.0 Hz frequency (0.76 ± 0.06). At other angles and frequencies, the PTRR showed no significant improvement.

The baseline MCdR without any stimulus (Model group) was measured at 0.29 ± 0.05, indicating that approximately one-third of mast cells were activated under resting conditions. The MCdR was also found to be dependent on acupuncture angle and frequency, reaching its peak with a 5-min duration, 180° rotation, and 1.0 Hz frequency [Fig. 4A2, 4B2]. Statistical analysis showed no significant differences in the total mast cell numbers across groups.

A correlation analysis between PTRR and MCdR was conducted, and the relevant data from all acupuncture parameter groups (N = 72) are plotted in [Fig. 4A3 and 4B3]. The PTRR and MCdR demonstrated a strong positive correlation, with very low P-values. These results suggest an intrinsic relationship between acupuncture-induced analgesia and mast cell degranulation.

### 2.4 Effect of mast cells

**Fig. 5:**
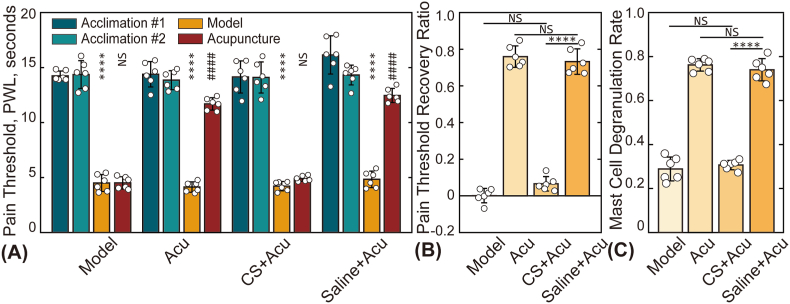
Influence of sodium cromoglycate (mast cell degranulation inhibitor). (A) Pain thresholds of animals in the three groups at four time points. Asterisks (∗) indicate statistical differences between the baseline pain threshold and post-model establishment values ($L_{0}$ vs. $L_{1}$); NS: not statistically significant; ∗∗∗∗: *p* < 0.0001. Hash symbols (#) denote statistical differences between pain thresholds before ($L_{1}$) and after ($L_{2}$) treatment; ####: *p* < 0.0001. (B) and (C) present the statistical results for the PTRR and MCdR, respectively.

In this study, sodium cromoglycate, a mast cell membrane stabilizer, was employed to investigate the role of mast cells in acupuncture-induced analgesia. The effects of sodium cromoglycate on PTRR and MCdR are presented in [Fig. 5]. As shown, injecting saline into the acupoint prior to acupuncture treatment had no significant effect on pain relief. In contrast, the injection of sodium cromoglycate effectively blocked the analgesic effects of acupuncture, resulting in a PTRR for the CS + Acu group approaching zero. Additionally, the MCdR in this group exhibited significant differences compared to the saline group.

### 2.5 Effect of histamine

**Fig. 6:**
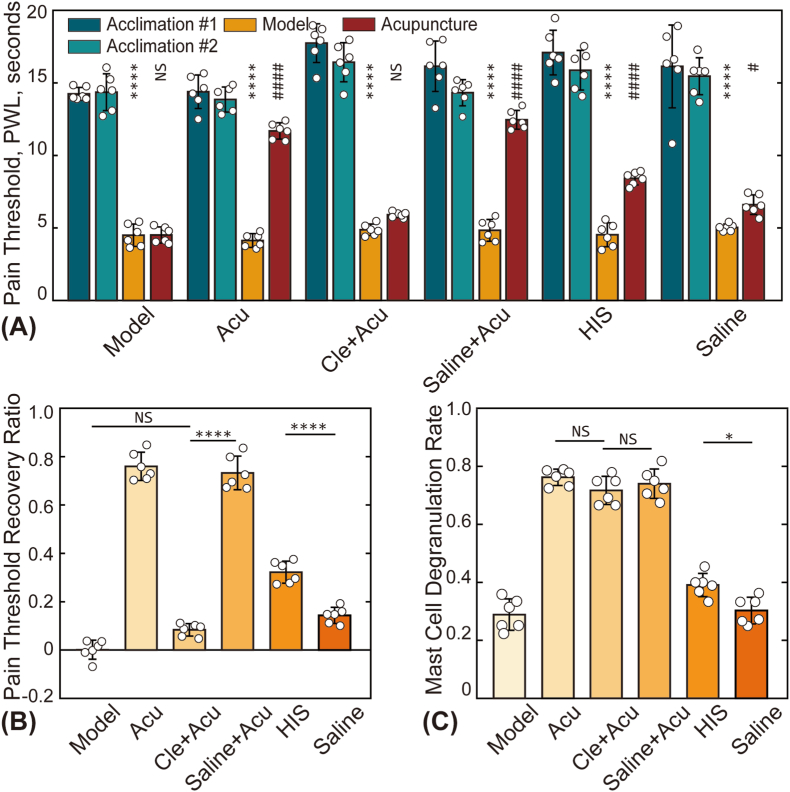
Comparison of pain thresholds and MCdR among various treatment groups. (A) Pain thresholds of animals in the Model group, robot-assisted acupuncture (specific subgroup of the RA group, Acu), Cle + Acu group, Saline + Acu group, HIS group, and Saline group at four time points. Asterisks (∗) indicate statistical differences between the baseline pain threshold and the values after model establishment ($L_{0}$ vs. $L_{1}$); NS: not statistically significant (*p* > 0.05); ∗: *p* < 0.05; ∗∗∗∗: *p* < 0.0001. Hash symbols (#) denote statistical differences between pain thresholds before ($L_{1}$) and after ($L_{2}$) treatment; #: *p* < 0.05; ####: *p* < 0.0001. (B) and (C) present the statistical results for PTRR and MCdR, respectively.

[Fig. 6] illustrates the effects of clemastine fumarate (an antihistamine) and direct histamine injection on PTRR and MCdR. As shown in [Fig. 6A and 6B], injection of saline into the acupoint prior to treatment did not affect pain relief. In contrast, administration of clemastine fumarate significantly obstructsed the analgesic effect, resulting in a PTRR close to zero for the Cle + Acu group, with no notable differences in MCdR across groups. Additionally, direct injection of histamine into the acupoint produced analgesic effects comparable to those of acupuncture, with a significant increase in MCdR compared to the saline group. These findings further emphasize the crucial role of histamine in mediating acupuncture-induced analgesic.

### 2.6 Effect of nerve distribution

**Fig. 7:**
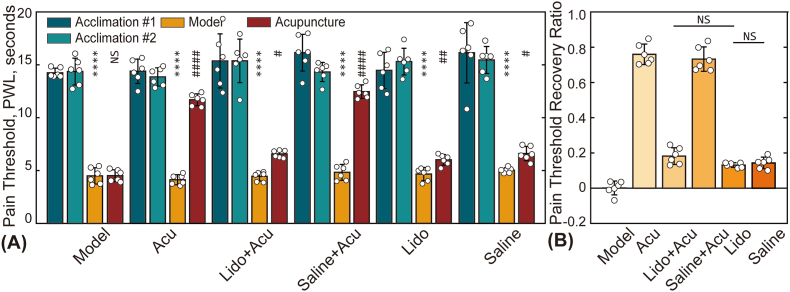
Comparison of pain thresholds among various treatment groups. (A) Pain thresholds of animals in the Model group, robot-arm assisted acupuncture (specific subgroup of the RA group, Acu), Lido + Acu group, Saline + Acu group, Lido group, and Saline group at four time points. Asterisks (∗) indicate statistically significant difference between the baseline pain threshold and the post-model establishment values ($L_{0}$ vs. $L_{1}$); ∗∗∗∗*p* < 0.0001. Hash symbols (#) denote statistically significant differences between pain thresholds before ($L_{1}$) and after ($L_{2}$) treatment; #*p* < 0.05; ##*p* < 0.01; ####*p* < 0.0001. (B) Presents the statistical results for the PTRR. No significant difference was observed between the groups (*p* > 0.05).

[Fig. 7] illustrates the changes in PTRR for the Lido + Acu group and the Lido group. The PTRR in the Lido + Acu group was significantly lower than that in the Acu group, indicating that inhibiting nerve function at the acupoint markedly reduces the analgesic effect of manual acupuncture. In contrast, the PTRR in the direct injection group was significantly lower than that in the saline group but higher than in the model group, suggesting that local injection of lidocaine still provides some analgesic effect, potentially by directly anesthetizing local nerves or tissues. These results indicate that lidocaine not only acts as a local anesthetic but also interferes with the pain modulation mechanisms associated with acupuncture, thereby confirming the important role of local neural pathways in the analgesic response.

## 3 Discussion

### 3.1 Influence of acupuncture parameters on analgesic effects

Understanding the relationship between acupuncture parameters, such as duration, intensity, manipulation techniques, and acupoint selection, and therapeutic outcomes is crucial for clinical practice and has garnered significant attention from researchers [26]. However, biomechanical studies on the effects of quantitative mechanical parameters remain limited. This study systematically investigates the effects of different acupuncture twisting parameters on analgesic outcomes, emphasizing the roles of mast cell activation, histamine (HIS) release, and neural conduction. Using an acute adjuvant-induced arthritis (AA) rat model, robot-assisted acupuncture twisting was performed with varying rotation angles (60°–360°) and frequencies (0.5–2.5 Hz). Multiple measurements of pain thresholds revealed that a combination of 180° rotation angle and a 1.0 Hz frequency produced the optimal analgesic effect [Fig. 3, Fig. 4], as quantified by the normalized PTRR (Eq. (1)). Moreover, this optimal analgesic effect was associated with higher MCdR values [Fig. 4].

The existence of optimal parameters is further supported by several other studies. Li et al. demonstrated that longer durations of manual acupuncture led to more significant effects on the central nervous system, highlighting the importance of needling time in therapeutic outcomes [27]. Liu et al. reported that manual acupuncture at 2 Hz was most effective for improving gastric motility in rats with atropine-induced gastric hypomotility, while lower (1 Hz) or higher frequencies (3 Hz and 4 Hz) were less effective [28]. Given that most experiments utilize manual acupuncture, accurately controlling various acupuncture parameters remains challenging for practitioners. Fortunately, this challenge is mitigated with robot-assisted acupuncture, which has been shown to be as effective as manual treatment [Fig. 1, Fig. 2]. The use of a robotic arm in this study not only reduces labor but also ensures that acupuncture frequency and amplitude are highly controllable, quantitatively precise, and consistently reliable.

### 3.2 The role of mast cell degranulation

The significance of mast cell degranulation in initiating the effects of acupuncture at local acupoints has been extensively documented [[29], [30], [31], [32]]. This study found that acupuncture effects are closely related to mast cell degranulation, as demonstrated by the injection of a mast cell membrane stabilizer at the acupuncture point [Fig. 5]. Furthermore, the strong correlation between the PTRR and MCdR observed in this study not only aligns with existing literature but also provides new insights into the mechanisms underlying acupuncture-induced analgesia. These findings suggest that the analgesic effects of acupuncture manipulation, particularly concerning rotation angle and frequency, may be closely linked to mast cell activation, potentially driven by variations in the degree of this activation.

The observed changes in mast cell degranulation under different acupuncture parameters imply that mast cells play a crucial role in mediating acupuncture-induced analgesia [Fig. 4]. Specifically, the extent of mast cell activation appears to influence the degree of pain relief, underscoring their pivotal role in the body's response to acupuncture stimulation. Moreover, mast cells are known to function as amplifiers in neuroimmune signaling networks by releasing various bioactive mediators such as histamine, tryptase, and cytokines [33]. These mediators can sensitize peripheral nociceptors or recruit additional immune cells, thereby amplifying either analgesic or pro-inflammatory responses depending on the local context [34]. This underscores the importance of selecting optimal acupuncture parameters to maximize therapeutic outcomes, as fine-tuning the intensity of stimulation may enhance mast cell activation and amplify analgesic effects. Together, these findings highlight future research directions, suggesting that deeper exploration of mast cell dynamics could refine acupuncture techniques and enhance our understanding of the biological mechanisms underlying acupuncture-induced analgesia.

However, the mechanisms by which different acupuncture frequencies and amplitudes alter mast cell degranulation remain unclear. One possible explanation lies in the biomechanics of needle-tissue interaction during acupuncture. Langevin et al. proposed that effective needle coupling with connective tissue is essential for therapeutic outcomes [[35], [36], [37]]. From a mechanotransduction perspective, the interaction between the acupuncture needle and connective tissue may lead to cytoskeletal deformation or integrin-mediated signaling in mast cells, thereby promoting intracellular calcium influx and subsequent degranulation [38]. Such biomechanical cues are increasingly recognized as critical initiators of cellular responses within the tissue microenvironment. Some researchers argue that this mechanical coupling is essential for transmitting external forces to the acupoint, generating local strain and stress that directly stimulate mast cells and trigger degranulation [17]. Therefore, needle-tissue interactions may be influenced by acupuncture parameters such as duration, angle, and frequency, as well as by additional factors including the mechanical properties of the acupoint, needle insertion depth, and manipulation techniques (e.g., lifting-thrusting or rotating). Different combinations of acupuncture frequency, angle, and amplitude may differentially affect the strength and pattern of mechanical coupling with the extracellular matrix, thereby altering the mechanical forces transmitted to mast cells and modulating their activation thresholds.

### 3.3 The role of histamine release

Histamine, a key substance released during mast cell degranulation [39], plays a significant role in acupuncture-induced analgesia. This biogenic amine is crucial for modulating both neurotransmission and immune responses by binding to various histamine receptors, such as H1 and H2 receptors. These interactions are involved in pain perception and contribute to the analgesic effects observed in acupuncture. Beyond its role in pain regulation, histamine also influences inflammatory responses and vascular permeability, which can further modulate pain transmission. Specifically, during acupuncture, histamine release can lead to local vasodilation and tissue inflammation [40], thereby affecting surrounding tissues and pain pathways, and potentially reducing pain perception by altering signal transmission [41].

Our findings demonstrate that pre-injection of antihistamines significantly blocks the analgesic effects of acupuncture [Fig. 6]. In the Cle + Acu group, although mast cells underwent extensive degranulation, the expected analgesic outcome was not achieved. These results suggest that mast cell degranulation alone may not be sufficient for acupuncture-induced pain relief if histamine signaling is inhibited. This observation underscores the importance of histamine as a mediator in the acupuncture process, as its inhibition by antihistamines negates the analgesic benefits, even in the presence of significant mast cell activity. Similar findings were reported by Huang et al., further reinforcing the critical role of histamine in mediating acupuncture-induced analgesia [42].

Moreover, the ability of histamine to influence both local and systemic inflammation highlights its dual role in facilitating and modulating the body's response to acupuncture. The local release of histamine not only promotes vasodilation, improving blood flow and tissue oxygenation, but also triggers a cascade of immune responses that may contribute to the therapeutic effects of acupuncture. However, when histamine receptors are blocked, these beneficial effects are diminished, indicating that histamine signaling is integral to the full spectrum of acupuncture's physiological impacts. Therefore, our study emphasizes the necessity of histamine in effectively initiating analgesia during acupuncture and suggests that further investigation into histamine's broader role could provide new insights for optimizing acupuncture techniques to enhance pain relief.

### 3.4 The role of neurotransmission

The analgesic effects of acupuncture are closely related not only to mast cell degranulation but also to local neural transmission mechanisms. Our study shows that local anesthetics, such as lidocaine, can significantly reduce the analgesic effects of acupuncture, further confirming the critical role of neural transmission in pain modulation during acupuncture [Fig. 7]. Mechanical stimulation from acupuncture may activate local sensory nerve fibers, particularly C-fibers and Aδ-fibers, thereby altering pain signaling pathways and reducing pain perception at the level of the central nervous system [43,44].

Specifically, acupuncture stimulation triggers the release of biochemical factors, such as histamine and neuropeptides, while also activating neural pathways that regulate nociceptor sensitivity. The mechanical stress generated by acupuncture can depolarize local neurons, initiating neural reflex arcs that transmit signals to the spinal cord and brain. This cascade ultimately modulates pain perception through a complex network of neural circuits. Thus, acupuncture-induced analgesia is not solely the result of chemical reactions but rather arises from a sophisticated interplay among the neural, immune, and endocrine systems [[45], [46], [47], [48]].

### 3.5 Limitations and prospects

Although this study provides quantitative data on the optimal angle and frequency for acupuncture rotation manipulation, these results cannot be directly extrapolated to clinical practice due to the inherent differences between animal models and humans, as well as variability in efficacy based on disease type, symptomatology, and acupoint selection. The primary contribution of this study lies in confirming the significance of acupuncture stimulation parameters on analgesic effects and in preliminarily elucidating the critical roles of mast cell degranulation and histamine signaling in acupuncture-induced analgesia. Future research should further investigate the relationship among acupuncture parameters, tissue biomechanics, and mast cell responses to enhance the clinical applicability of findings.

## 4 Materials and methods

### 4.1 Animals

This study utilized 114 healthy adult male Sprague Dawley (SD) rats (SPF grade, weighing 200 ± 20 g). The rats were housed in the Animal Experiment Center of Shanghai University of Traditional Chinese Medicine under controlled conditions (22–25 °C, 50 %–60 % humidity, and a 12-h light/dark cycle).

The animals were randomly divided into the following groups: the model group (Model), the needle-retaining group (NR), the manual acupuncture group (MA), the robot-assisted acupuncture group (RA, further subdivided into 9 subgroups), the Cromolyn Sodium (a mast cell membrane stabilizer) combined with acupuncture group (CS + Acu), the Clemastine fumarate combined with acupuncture group (Cle + Acu), the Histamine group (HIS), the Lidocaine hydrochloride combined with acupuncture group (Lido + Acu), the Lidocaine hydrochloride group (Lido), the saline combined with acupuncture group (Saline + Acu), and the saline group (Saline). Each group or subgroup consisted of six rats.

### 4.2 Acute adjuvant arthritis model (AA model)

The current study employed the widely used acute adjuvant arthritis (AA) rat model for acupuncture research [21]. The model was established by injecting 200 μL of complete Freund's adjuvant (CFA) into the left ankle joint cavity of the animal. Local ankle swelling, characterized by noticeable redness and increased volume, was observed 48 h after CFA injection. Additionally, significant behavioral changes, such as altered gait and reduced activity, were evident.

### 4.3 Behavioral tests of pain threshold

Thermal pain threshold measurements were utilized to assess the animal's pain sensitivity. Each animal was acclimated to the testing environment by being placed in the testing cage for approximately 30 min daily over three consecutive days prior to the experiment. During the test, a radiant heat beam (IITC336G, IITC Life Science, USA) was directed at the left paw, and the latency time (in seconds) before paw withdrawal was measured to estimate the thermal pain threshold, defined as the paw withdrawal latency (PWL). To prevent potential tissue damage, the maximum heating time was limited to 20 s. Each test was repeated three times at 5-min intervals, and the average value was recorded as the final pain threshold.

Each animal underwent four behavioral tests at different time points throughout the experiment: two tests during the acclimation period (Day −1 and Day 0, with the averaged results noted as $L_{0}$), one test after the establishment of the AA model (Day 2, denoted as $L_{1}$), and one test after treatment (Day 2, post-treatment, denoted as $L_{2}$). To eliminate individual differences in baseline pain threshold, a dimensionless index termed the pain threshold recovery ratio (PTRR) was calculated as follows:

(1) $$PTRR=\frac{L_{2}-L_{1}}{L_{0}-L_{1}}\text{,}$$

where $L_{0}-L_{1}$ represents the drop in pain threshold after modeling, indicating increased sensitivity to heat stimulus, and $L_{2}-L_{1}$ estimates the recovery of the pain threshold after treatment. PTRR is defined as the ratio of recovery to drop, serving as an evaluation metric for analgesic effect. A PTRR close to 0 indicates ineffective treatment, while a PTRR approaching 1 signifies recovery of the pain threshold to baseline levels, indicating effective treatment.

### 4.4 Treatment interventions

[Table S1] provides an overview of the different treatment interventions applied across all groups and subgroups in this study. Animals in the RA groups were treated at the ST36 acupoint, utilizing a stepper motor mounted on a robot-arm (RM65, Realman, China) to precisely control various combinations of stimulus parameters (angle and frequency) for each subgroup [Fig. 1A].

The MA group received twirling-rotating acupuncture with a duration of 5 min, an angle of approximately 180°, and a frequency of about 1.0 Hz. No interventions were applied to the Model group. In the NR group, an acupuncture needle was inserted into the acupoint without subsequent manipulation. In the CS + Acu group, an intramuscular injection of Cromolyn Sodium (100 μL, 2 mg per rat) was administered into the ST36 acupoint 20 min prior to the acupuncture treatment. Similarly, the Cle + Acu group received an injection of Clemastine fumarate (100 μL, 0.18 mg). The His group was administered Histamine (100 μL, 1 mg) 25 min before pain threshold measurement. Lidocaine hydrochloride (100 μL, 0.8 %) was administered to both the Lido + Acu and Lido groups, 20 and 25 min prior to acupuncture or measurement, respectively. The Saline + Acu and Saline groups received 100 μL of saline under similar conditions.

Both manual and robot-assisted acupuncture were performed at the ST36 point (approximately 5 mm below the fibula head, on the lateral side behind the knee joint) [49]. Fine and sharp steel needles (diameter 0.3 mm, length 25 mm, Huatuo, China) were utilized for acupuncture stimulation. To minimize inter-animal variability and motion artifacts, each rat was fitted with a light-shielding hood during treatment to reduce stress-induced activity. Animals were placed in the prone position, with the left hind limb gently extended and fixed using adhesive tape, ensuring reproducible limb positioning and minimizing movement during needling. To further standardize the procedure, needle insertion depth was controlled: acupuncture needles (25 mm body length) were inserted to a depth of 7 mm (leaving 18 mm exposed), and the insertion angle was maintained as perpendicular as possible to the skin surface. Minor angular deviations were unavoidable but were minimized through consistent positioning.

To ensure blinding and data consistency, both the pain threshold assessments and mast cell degranulation analyses were performed by the same trained experimenter, who was blinded to group allocation throughout the procedures.

### 4.5 Verification of robot-assisted acupuncture

To verify the precise control of the robot arm, an image recognition method was employed to measure the angular displacement of the needle movement driven by the robotic system. The results demonstrated that both rotation angle and frequency could be effectively controlled, with a relative error of less than 5 % for both parameters [Fig. 1B]. To further ensure that the programmed rotation in free space accurately reflects rotation within biological tissue, both technical feasibility and motor capacity were carefully considered. Real-time angle monitoring under biological loading was technically challenging due to tissue occlusion and restricted viewing angles during rotation. Therefore, angular assessments were conducted under no-load conditions using image recognition. In addition, the stepper motor (Model: 42CM08, Leadshine, China, holding torque 0.8 N m, operational torque >0.6 N m at 500 rpm) used in our system provides a torque output much greater than the load typically encountered during acupuncture in rats (∼10–15 mN mm, as reported by Zhang et al., [50]). Given this margin, the angular output measured under unloaded conditions is considered functionally equivalent to that under biological load, thereby supporting the validity of free-space angle measurements for in vivo applications. Moreover, needle displacement under manual control was measured using the same method, providing a quantitative estimation of stimulus angle and frequency for the MA group [Fig. 1C].

### 4.6 Estimation of mast cell degranulation rate (MCdR)

Following euthanasia via an overdose of CO_2_ anesthesia, tissue samples were immediately collected and fixed in 4 % paraformaldehyde. The tissues were then dehydrated, embedded in paraffin, and sectioned into 5 μm-thick slices. Sections were stained with toluidine blue, allowing clear distinction of mast cells under an optical microscope (NTB900-FL, Ningbo Yongxin Optical, China). Un-degranulated mast cells appeared as circular, oval, or spindle-shaped isolated dots, whereas degranulated mast cells exhibited dispersed purple granules around the cell body [Fig. S1]. To calculate the MCdR, six sections from each animal were randomly selected (three containing muscle tissue and three containing skin tissue). From each section, one or two representative fields of view (FOVs) were chosen under the microscope, resulting in a total of six FOVs per animal (three from skin, three from muscle). All selected FOVs contained at least one mast cell to ensure biological relevance and minimize sampling error due to mast cell sparsity in certain regions. This approach enabled coverage of both superficial and deeper tissue layers, mitigating the impact of regional variability in mast cell distribution. In the selected FOVs, the total number of degranulated and un-degranulated mast cells were counted for each animal, and the MCdR was calculated as the proportion of degranulated cells to total cell numbers. To ensure methodological consistency and statistical reliability, six FOVs were analyzed per animal, in accordance with standard practices reported in related studies [51,52]. In our dataset, the total number of mast cells per rat typically ranged from 11 to 43, which aligns with previously reported ranges (10–33, [53]) and further supports the validity of our sampling strategy.

### 4.7 Statistical analysis

All pain threshold results and MCdR data were analyzed using the Statistics and Machine Learning Toolbox in MATLAB (R2023a, MathWorks, USA). Data are expressed as mean ± standard deviation. Group comparisons were conducted using one-way analysis of variance (ANOVA), followed by Tukey's post-hoc tests for pairwise comparisons. A P-value <0.05 was considered statistically significant.

## 5 Conclusions

This study demonstrates that acupuncture parameters, specifically rotation angle and frequency, significantly influence analgesic outcomes. The optimal combination of a 180° rotation angle and a 1.0 Hz frequency was identified as the most effective for achieving acupuncture-induced analgesia in AA rat models. This parameter-dependent therapeutic effect is mediated by mast cell degranulation and the subsequent release of bioactive substances, particularly histamine, and is likely associated with local mechanical interactions between the acupuncture needle and surrounding tissue. Local histamine injections replicated the analgesic effects induced by acupuncture, whereas H1 receptor antagonists and lidocaine attenuated these effects. These findings further underscore that the key mechanism underlying acupuncture-induced analgesia is mediated through histamine release and local neural conduction.

## Author contributions

Conceptualization, Y.Y., W.Y. and Z-Q.H.; methodology, Y-X.W., Y–H.L., X.Q. and Y–C.L.; software, X.Q. and Y–C.L.; validation, Y.Y. and X.Q.; formal analysis, Y.Y. and Y-X.W.; writing—original draft preparation, Y.Y. and Y-X.W.; writing—review and editing, Y.Y. and W.Y.; visualization, Y.Y. and Y-X.W.; supervision, W.Y.; funding acquisition, Y.Y., W.Y. and Z-Q.H. All authors have read and agreed to the published version of the manuscript.

## Institutional review board statement

The experiment was in accordance with the Animal Management Rules of the Ministry of Science and Technology of the People's Republic of China for experimental care and use of animals, and the research protocol was approved by the Animals Ethics Committee of Shanghai University of Traditional Chinese Medicine (Protocol No. PZSHUTCM2401150001).

## Funding

This research was funded by 10.13039/501100001809National Natural Science Foundation of China, grant number 82305416 (Yu), 12172092 (Yao), 82174488 (Yao). Additional support was provided by the 10.13039/501100003399Shanghai Municipal Science and Technology Commission, grant number 23YF1418300 (Yu) and the University Research Fund of Shanghai University of Medicine & Health Sciences, grant number SSF-23-04-004 (Yu). New Quality Specialty Clinical Medicine Construction Funding of Peak Plateau Discipline Construction of Shanghai Pudong New Area 2025-PWXZ-17 (Huang).

## Conflict of interests

The authors declare no conflict of interests.

## Data Availability

A brief report of the original data was included in the supplementary material. More information is available upon reasonable request to the first and corresponding authors.
