# Supplementary material for: Influence of acupuncture twisting parameters on analgesic effects mediated by mast cells in AA rat models
Source: Biomed J. 2025 Jun 4;49(1):100876. doi: 10.1016/j.bj.2025.100876 (PMC12860710; doi:10.1016/j.bj.2025.100876)
Supplement: Multimedia component 1 [file mmc1.docx]

Supplementary Material

Influence of acupuncture twisting parameters on analgesic effects mediated by mast cells in AA rat models


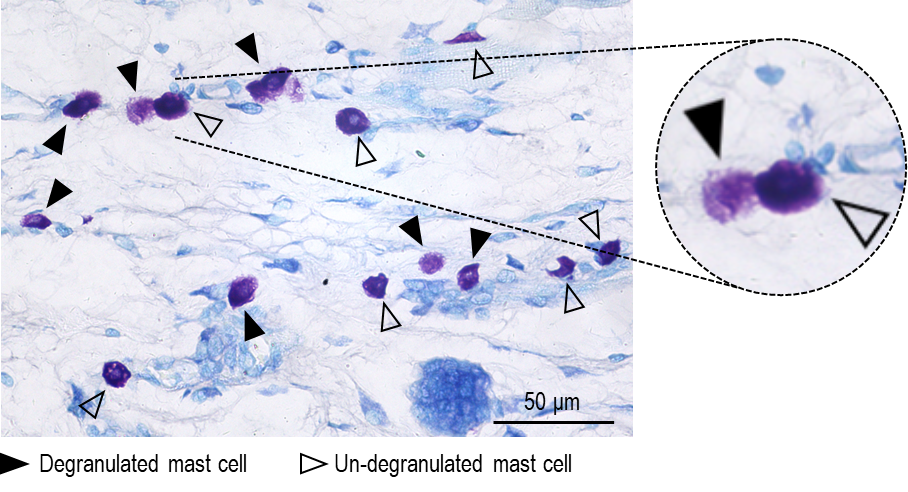


**Figure S1**. Example section of stained mast cells within the acupoint tissues. The mast cells appear purple and can be classified into two states: degranulated and intact. Intact mast cells exhibit relatively clear cell borders, whereas degranulated mast cells are characterized by numerous tiny particles or granules distributed around the cell body.

**
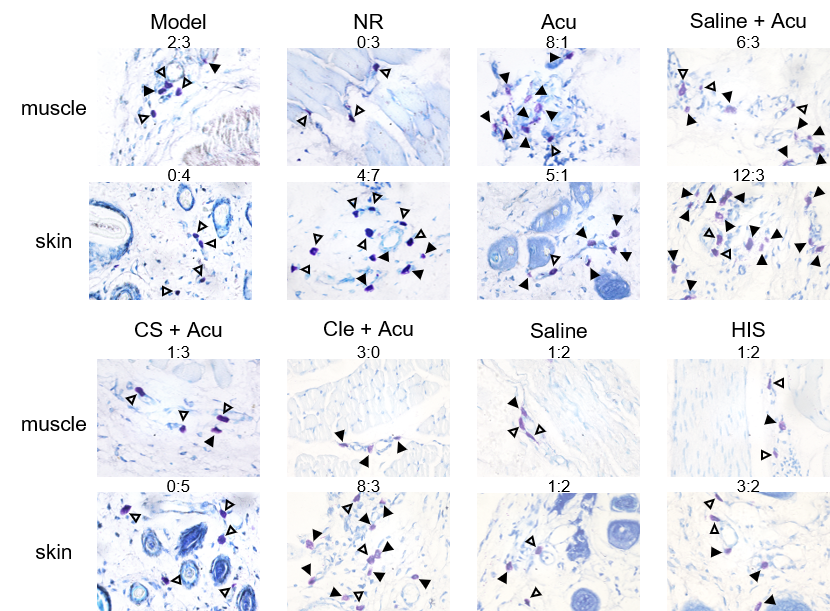
**

**Figure S2**. Representative toluidine blue-stained images of the major experimental groups. Solid triangles indicate degranulated mast cells, while hollow triangles denote intact mast cells.The degranulation ratio is shown above each image.

**Table S1.** An overview of treatment interventions of different animal groups

| **Group** | **Interventions** | **Subgroups** | **Stimulus parameters** | | |
| --- | --- | --- | --- | --- | --- |
|  |  |  | **Duration, min** | **Angle, degree** | **Frequency, Hz** |
| **Model** | No intervention | - | - | - | - |
| **NR** | Inserting the needle, without stimulus, for 5 min | - | 5 | - | - |
| **MA** | Manual acupuncture treatment | - | 5 | 180 | 1.0 |
| **RA** | Robot-arm assisted acupuncture treatment (ST36) | 1^+,#^ | 5 | 180 | 1.0 |
|  |  | 2^+^ | 5 | 60 | 1.0 |
|  |  | 3^+^ | 5 | 90 | 1.0 |
|  |  | 4^+^ | 5 | 270 | 1.0 |
|  |  | 5^+^ | 5 | 360 | 1.0 |
|  |  | 6^#^ | 5 | 180 | 0.5 |
|  |  | 7^#^ | 5 | 180 | 1.5 |
|  |  | 8^#^ | 5 | 180 | 2.0 |
|  |  | 9^#^ | 5 | 180 | 2.5 |
| **CS + Acu** | Pre-injection of Cromolyn Sodium before acupuncture | - | 5 | 180 | 1.0 |
| **Cle + Acu** | Pre-injection of Clemastine fumarate before acupuncture | - | 5 | 180 | 1.0 |
| **HIS** | Injection of histamine | - | - | - | - |
| **Lido + Acu** | Pre-injection of Lidocaine hydrochloride before acupuncture | - | 5 | 180 | 1.0 |
| **Lido** | Injection of Lidocaine hydrochloride | - | - | - | - |
| **Saline + Acu** | Pre-injection of saline before acupuncture | - | 5 | 180 | 1.0 |
| **Saline** | Injection of saline | - | - | - | - |
|  | + These subgroups were adopted to analyze the influence of acupuncture angle.  # These subgroups were adopted to analyze the influence of acupuncture frequency. | | | | |

**Table S2.** Original data of this study (N=114 rats)

| **Group** | **Subgroup** | **Animal** | **Thermal pain threshold, PWL, seconds** | | | | | **PTRR** | **MCdR** |
| --- | --- | --- | --- | --- | --- | --- | --- | --- | --- |
|  |  |  | Acclimation  Day -2 | Acclimation  Day -1 | Baseline $L_{0}$ | Model $L_{1}$  Day 2 | Treatment $L_{2}$, Day 2 |  |  |
| **RA** | **Subgroup 1**  5 min  180°  1.0 Hz | #1 | 14.27 | 13.41 | 13.84 | 4.42 | 12.41 | 0.85 | 15/19=0.79 |
|  |  | #2 | 13.83 | 14.58 | 14.21 | 4.43 | 11.84 | 0.76 | 21/29=0.72 |
|  |  | #3 | 14.65 | 12.82 | 13.74 | 3.57 | 10.99 | 0.73 | 13/17=0.76 |
|  |  | #4 | 12.50 | 13.07 | 12.79 | 3.86 | 11.09 | 0.81 | 19/26=0.73 |
|  |  | #5 | 15.42 | 14.87 | 15.15 | 4.77 | 12.13 | 0.71 | 22/28=0.79 |
|  |  | #6 | 15.66 | 14.38 | 15.02 | 3.74 | 11.67 | 0.70 | 14/18=0.78 |
|  |  | **Mean** | **14.39** | **13.85** | **14.12** | **4.13** | **11.69** | **0.76** | **0.76** |
|  |  | **S.D.** | **1.15** | **0.86** | **0.88** | **0.47** | **0.56** | **0.06** | **0.03** |
|  | **Subgroup 2**  5 min  60°  1.0 Hz | #1 | 16.42 | 14.14 | 15.28 | 4.80 | 7.85 | 0.29 | 8/20=0.40 |
|  |  | #2 | 12.58 | 11.74 | 12.16 | 3.95 | 5.46 | 0.18 | 4/13=0.31 |
|  |  | #3 | 13.25 | 12.88 | 13.06 | 3.55 | 5.29 | 0.18 | 9/21=0.43 |
|  |  | #4 | 13.93 | 13.36 | 13.65 | 4.92 | 7.73 | 0.32 | 12/31=0.39 |
|  |  | #5 | 12.01 | 11.68 | 11.84 | 4.15 | 5.44 | 0.17 | 10/24=0.42 |
|  |  | #6 | 14.81 | 13.88 | 14.34 | 4.78 | 6.49 | 0.18 | 3/11=0.27 |
|  |  | **Mean** | **13.83** | **12.95** | **13.39** | **4.36** | **6.38** | **0.22** | **0.37** |
|  |  | **S.D.** | **1.60** | **1.05** | **1.31** | **0.56** | **1.17** | **0.07** | **0.06** |
|  | **Subgroup 3**  5 min  90°  1.0 Hz | #1 | 13.90 | 12.74 | 13.32 | 3.70 | 6.91 | 0.33 | 9/14=0.64 |
|  |  | #2 | 15.30 | 13.99 | 14.64 | 3.87 | 6.16 | 0.21 | 9/18=0.50 |
|  |  | #3 | 14.27 | 14.05 | 14.16 | 3.86 | 6.87 | 0.29 | 13/21=0.62 |
|  |  | #4 | 16.20 | 13.79 | 15.00 | 3.39 | 6.71 | 0.29 | 8/13=0.62 |
|  |  | #5 | 16.51 | 14.80 | 15.65 | 3.45 | 8.01 | 0.37 | 8/14=0.57 |
|  |  | #6 | 14.20 | 12.71 | 13.46 | 3.90 | 7.67 | 0.39 | 9/18=0.50 |
|  |  | **Mean** | **15.06** | **13.68** | **14.37** | **3.69** | **7.05** | **0.32** | **0.57** |
|  |  | **S.D.** | **1.11** | **0.81** | **0.90** | **0.23** | **0.67** | **0.07** | **0.06** |
|  | **Subgroup 4**  5 min  270°  1.0 Hz | #1 | 14.14 | 12.07 | 13.11 | 3.89 | 6.71 | 0.31 | 14/23=0.61 |
|  |  | #2 | 12.78 | 13.00 | 12.89 | 3.81 | 8.47 | 0.51 | 7/13=0.54 |
|  |  | #3 | 14.31 | 12.95 | 13.63 | 4.24 | 7.38 | 0.33 | 14/22=0.64 |
|  |  | #4 | 14.98 | 12.92 | 13.95 | 3.75 | 9.17 | 0.53 | 8/14=0.57 |
|  |  | #5 | 12.47 | 15.39 | 13.93 | 4.01 | 8.67 | 0.47 | 10/17=0.59 |
|  |  | #6 | 15.11 | 14.39 | 14.75 | 5.22 | 9.55 | 0.45 | 20/39=0.51 |
|  |  | **Mean** | **13.97** | **13.45** | **13.71** | **4.15** | **8.32** | **0.43** | **0.58** |
|  |  | **S.D.** | **1.11** | **1.21** | **0.67** | **0.55** | **1.08** | **0.09** | **0.05** |
|  | **Subgroup 5**  5 min  360°  1.0 Hz | #1 | 14.05 | 13.49 | 13.77 | 4.57 | 7.45 | 0.31 | 7/15=0.47 |
|  |  | #2 | 13.42 | 12.86 | 13.14 | 4.50 | 7.74 | 0.38 | 10/21=0.48 |
|  |  | #3 | 16.65 | 15.82 | 16.23 | 4.85 | 6.60 | 0.15 | 9/17=0.53 |
|  |  | #4 | 11.36 | 11.87 | 11.61 | 4.88 | 7.31 | 0.36 | 7/17=0.41 |
|  |  | #5 | 14.97 | 13.28 | 14.12 | 5.28 | 6.90 | 0.18 | 8/15=0.53 |
|  |  | #6 | 12.71 | 13.84 | 13.27 | 5.01 | 7.68 | 0.32 | 10/19=0.53 |
|  |  | **Mean** | **13.86** | **13.52** | **13.69** | **4.85** | **7.28** | **0.28** | **0.49** |
|  |  | **S.D.** | **1.83** | **1.31** | **1.51** | **0.29** | **0.45** | **0.09** | **0.05** |
|  | **Subgroup 6**  5 min  180°  0.5 Hz | #1 | 14.95 | 12.79 | 13.87 | 4.53 | 6.93 | 0.26 | 9/21=0.43 |
|  |  | #2 | 14.24 | 15.58 | 14.91 | 4.59 | 5.73 | 0.11 | 7/15=0.47 |
|  |  | #3 | 14.71 | 14.02 | 14.37 | 4.45 | 5.99 | 0.16 | 11/25=0.44 |
|  |  | #4 | 14.56 | 12.16 | 13.36 | 4.15 | 6.57 | 0.26 | 7/16=0.44 |
|  |  | #5 | 14.29 | 11.59 | 12.94 | 3.83 | 5.21 | 0.15 | 6/13=0.46 |
|  |  | #6 | 12.03 | 12.75 | 12.39 | 3.34 | 5.75 | 0.27 | 6/14=0.43 |
|  |  | **Mean** | **14.13** | **13.15** | **13.64** | **4.15** | **6.03** | **0.20** | **0.44** |
|  |  | **S.D.** | **1.06** | **1.44** | **0.93** | **0.49** | **0.62** | **0.07** | **0.02** |
|  | **Subgroup 7**  5 min  180°  1.5 Hz | #1 | 18.45 | 12.92 | 15.69 | 3.64 | 6.92 | 0.27 | 10/20=0.50 |
|  |  | #2 | 14.30 | 13.95 | 14.13 | 4.08 | 8.89 | 0.48 | 11/19=0.58 |
|  |  | #3 | 12.77 | 12.28 | 12.53 | 4.31 | 7.60 | 0.40 | 8/12=0.67 |
|  |  | #4 | 15.23 | 13.15 | 14.19 | 4.81 | 7.49 | 0.29 | 14/24=0.58 |
|  |  | #5 | 15.81 | 14.77 | 15.29 | 3.81 | 6.94 | 0.27 | 8/14=0.57 |
|  |  | #6 | 15.79 | 13.41 | 14.60 | 3.81 | 7.39 | 0.33 | 10/16=0.63 |
|  |  | **Mean** | **15.39** | **13.41** | **14.40** | **4.08** | **7.54** | **0.34** | **0.59** |
|  |  | **S.D.** | **1.89** | **0.87** | **1.11** | **0.43** | **0.72** | **0.08** | **0.06** |
|  | **Subgroup 8**  5 min  180°  2.0 Hz | #1 | 15.25 | 14.25 | 14.75 | 4.23 | 6.31 | 0.20 | 7/14=0.50 |
|  |  | #2 | 15.40 | 15.81 | 15.60 | 5.04 | 8.52 | 0.33 | 8/17=0.47 |
|  |  | #3 | 13.54 | 14.62 | 14.08 | 3.89 | 7.06 | 0.31 | 8/16=0.50 |
|  |  | #4 | 12.71 | 11.59 | 12.15 | 3.96 | 5.44 | 0.18 | 6/13=0.46 |
|  |  | #5 | 13.52 | 11.51 | 12.52 | 4.49 | 6.98 | 0.31 | 7/14=0.50 |
|  |  | #6 | 14.01 | 13.03 | 13.52 | 4.52 | 7.74 | 0.36 | 10/22=0.45 |
|  |  | **Mean** | **14.07** | **13.47** | **13.77** | **4.35** | **7.01** | **0.28** | **0.48** |
|  |  | **S.D.** | **1.06** | **1.73** | **1.32** | **0.43** | **1.07** | **0.07** | **0.02** |

**Table S2 (Continued).** Original data of this study (N=114 rats)

| **Group** | **Subgroup** | **Animal** | **Thermal pain threshold, PWL, seconds** | | | | | **PTRR** | **MCdR** |
| --- | --- | --- | --- | --- | --- | --- | --- | --- | --- |
|  |  |  | Acclimation  Day -2 | Acclimation  Day -1 | Baseline $L_{0}$ | Model $L_{1}$  Day 2 | Treatment $L_{2}$, Day 2 |  |  |
| **RA** | **Subgroup 9**  5 min  180°  2.5 Hz | #1 | 14.82 | 12.88 | 13.85 | 4.53 | 7.02 | 0.27 | 8/22=0.36 |
|  |  | #2 | 16.75 | 17.33 | 17.04 | 3.78 | 6.57 | 0.21 | 5/14=0.36 |
|  |  | #3 | 15.16 | 14.74 | 14.95 | 4.73 | 7.67 | 0.29 | 8/21=0.38 |
|  |  | #4 | 13.36 | 13.27 | 13.32 | 4.17 | 6.72 | 0.28 | 5/13=0.38 |
|  |  | #5 | 14.10 | 16.08 | 15.09 | 4.37 | 6.55 | 0.20 | 6/14=0.43 |
|  |  | #6 | 13.96 | 13.50 | 13.73 | 4.85 | 7.07 | 0.25 | 4/16=0.25 |
|  |  | **Mean** | **14.69** | **14.63** | **14.66** | **4.40** | **6.93** | **0.25** | **0.36** |
|  |  | **S.D.** | **1.20** | **1.76** | **1.36** | **0.39** | **0.42** | **0.04** | **0.06** |
| **Model** | **-** | #1 | 14.07 | 16.06 | 15.07 | 4.15 | 4.13 | 0.00 | 4/16=0.25 |
|  |  | #2 | 13.90 | 14.56 | 14.23 | 3.65 | 4.04 | 0.04 | 5/14=0.36 |
|  |  | #3 | 14.85 | 12.72 | 13.79 | 3.75 | 3.91 | 0.02 | 5/16=0.31 |
|  |  | #4 | 14.00 | 13.09 | 13.55 | 4.72 | 5.03 | 0.04 | 4/16=0.25 |
|  |  | #5 | 13.91 | 15.25 | 14.58 | 5.29 | 5.20 | -0.01 | 4/18=0.22 |
|  |  | #6 | 14.77 | 14.48 | 14.63 | 5.43 | 4.80 | -0.07 | 5/15=0.33 |
|  |  | **Mean** | **14.25** | **14.36** | **14.31** | **4.50** | **4.52** | **0.00** | **0.29** |
|  |  | **S.D.** | **0.44** | **1.27** | **0.57** | **0.77** | **0.56** | **0.04** | **0.05** |
| **NR** | **-** | #1 | 12.67 | 10.41 | 11.54 | 3.84 | 5.15 | 0.17 | 5/14=0.36 |
|  |  | #2 | 13.06 | 12.45 | 12.76 | 4.85 | 6.52 | 0.21 | 5/15=0.33 |
|  |  | #3 | 13.79 | 10.04 | 11.92 | 4.26 | 5.98 | 0.22 | 6/19=0.32 |
|  |  | #4 | 14.20 | 12.29 | 13.25 | 4.78 | 5.79 | 0.12 | 7/21=0.33 |
|  |  | #5 | 13.27 | 11.72 | 12.50 | 5.05 | 6.07 | 0.14 | 10/26=0.38 |
|  |  | #6 | 16.63 | 13.65 | 15.14 | 4.04 | 5.50 | 0.13 | 7/24=0.29 |
|  |  | **Mean** | **13.94** | **11.76** | **12.85** | **4.47** | **5.84** | **0.17** | **0.34** |
|  |  | **S.D.** | **1.42** | **1.35** | **1.27** | **0.49** | **0.47** | **0.04** | **0.03** |
| **MA** | ~5 min  ~180°  ~1.0 Hz | #1 | 11.36 | 11.18 | 11.27 | 4.71 | 9.92 | 0.79 | 20/23=0.87 |
|  |  | #2 | 11.59 | 13.14 | 12.37 | 4.11 | 10.90 | 0.82 | 15/20=0.75 |
|  |  | #3 | 13.71 | 12.90 | 13.31 | 4.48 | 9.49 | 0.57 | 20/24=0.83 |
|  |  | #4 | 12.55 | 13.56 | 13.06 | 4.18 | 10.41 | 0.70 | 18/21=0.86 |
|  |  | #5 | 13.14 | 13.62 | 13.38 | 4.28 | 10.31 | 0.66 | 16/24=0.67 |
|  |  | #6 | 9.78 | 12.36 | 11.07 | 4.83 | 9.93 | 0.82 | 13/19=0.68 |
|  |  | **Mean** | **12.02** | **12.79** | **12.41** | **4.43** | **10.16** | **0.73** | **0.78** |
|  |  | **S.D.** | **1.42** | **0.91** | **1.02** | **0.29** | **0.49** | **0.10** | **0.09** |
| **CS**  **+**  **Acu** | ~5 min  ~180°  ~1.0 Hz | #1 | 15.59 | 12.09 | 13.84 | 4.09 | 4.68 | 0.06 | 5/16=0.31 |
|  |  | #2 | 14.68 | 16.03 | 15.36 | 4.59 | 5.16 | 0.05 | 7/22=0.32 |
|  |  | #3 | 14.02 | 13.68 | 13.85 | 3.84 | 4.62 | 0.08 | 3/11=0.27 |
|  |  | #4 | 11.95 | 14.20 | 13.08 | 4.60 | 4.93 | 0.04 | 7/24=0.29 |
|  |  | #5 | 15.63 | 15.28 | 15.45 | 3.61 | 5.24 | 0.14 | 5/16=0.31 |
|  |  | #6 | 13.00 | 13.32 | 13.16 | 4.53 | 4.77 | 0.03 | 5/15=0.33 |
|  |  | **Mean** | **14.14** | **14.10** | **14.12** | **4.21** | **4.90** | **0.07** | **0.31** |
|  |  | **S.D.** | **1.46** | **1.41** | **1.05** | **0.43** | **0.26** | **0.04** | **0.02** |
| **Cle**  **+**  **Acu** | ~5 min  ~180°  ~1.0 Hz | #1 | 15.33 | 14.19 | 14.76 | 5.47 | 5.99 | 0.06 | 15/20=0.75 |
|  |  | #2 | 18.97 | 15.75 | 17.36 | 4.53 | 5.65 | 0.09 | 32/41=0.78 |
|  |  | #3 | 17.21 | 17.97 | 17.59 | 4.89 | 6.19 | 0.10 | 14/21=0.67 |
|  |  | #4 | 18.30 | 16.75 | 17.53 | 4.40 | 5.86 | 0.11 | 12/16=0.75 |
|  |  | #5 | 17.92 | 17.48 | 17.70 | 5.15 | 5.74 | 0.05 | 8/12=0.67 |
|  |  | #6 | 18.71 | 16.40 | 17.55 | 4.79 | 6.02 | 0.10 | 9/13=0.69 |
|  |  | **Mean** | **17.74** | **16.42** | **17.08** | **4.87** | **5.91** | **0.08** | **0.72** |
|  |  | **S.D.** | **1.33** | **1.35** | **1.14** | **0.40** | **0.20** | **0.03** | **0.05** |
| **HIS** | **-** | #1 | 19.47 | 16.85 | 18.16 | 3.48 | 8.80 | 0.36 | 4/12=0.33 |
|  |  | #2 | 16.87 | 16.66 | 16.76 | 3.73 | 8.63 | 0.38 | 7/19=0.37 |
|  |  | #3 | 16.68 | 14.60 | 15.64 | 5.13 | 8.05 | 0.28 | 4/10=0.40 |
|  |  | #4 | 18.14 | 17.53 | 17.83 | 5.59 | 8.92 | 0.27 | 7/18=0.39 |
|  |  | #5 | 16.37 | 15.53 | 15.95 | 4.33 | 8.38 | 0.35 | 5/11=0.45 |
|  |  | #6 | 15.01 | 14.08 | 14.54 | 4.89 | 7.75 | 0.30 | 8/20=0.40 |
|  |  | **Mean** | **17.09** | **15.87** | **16.48** | **4.53** | **8.42** | **0.32** | **0.39** |
|  |  | **S.D.** | **1.54** | **1.36** | **1.38** | **0.82** | **0.45** | **0.05** | **0.04** |
| **Lido**  **+**  **Acu** | ~5 min  ~180°  ~1.0 Hz | #1 | 16.93 | 15.11 | 16.02 | 4.79 | 6.26 | 0.13 | **-** |
|  |  | #2 | 19.36 | 15.91 | 17.64 | 4.88 | 6.94 | 0.16 | **-** |
|  |  | #3 | 13.23 | 11.67 | 12.45 | 3.93 | 6.03 | 0.25 | **-** |
|  |  | #4 | 14.55 | 17.92 | 16.24 | 4.59 | 6.83 | 0.19 | **-** |
|  |  | #5 | 15.75 | 15.51 | 15.63 | 4.65 | 6.16 | 0.14 | **-** |
|  |  | #6 | 12.37 | 16.13 | 14.25 | 3.87 | 6.21 | 0.23 | **-** |
|  |  | **Mean** | **15.37** | **15.38** | **15.37** | **4.45** | **6.40** | **0.18** | **-** |
|  |  | **S.D.** | **2.56** | **2.06** | **1.80** | **0.44** | **0.38** | **0.05** | **-** |

**Table S2 (Continued).** Original data of this study (N=114 rats)

| **Group** | **Subgroup** | **Animal** | **Thermal pain threshold, PWL, seconds** | | | | | **PTRR** | **MCdR** |
| --- | --- | --- | --- | --- | --- | --- | --- | --- | --- |
|  |  |  | Acclimation  Day -2 | Acclimation  Day -1 | Baseline $L_{0}$ | Model $L_{1}$  Day 2 | Treatment $L_{2}$, Day 2 |  |  |
| **Lido** | - | #1 | 14.29 | 15.55 | 14.92 | 4.81 | 5.96 | 0.11 | **-** |
|  |  | #2 | 16.09 | 13.64 | 14.87 | 5.00 | 6.38 | 0.14 | **-** |
|  |  | #3 | 14.96 | 15.23 | 15.10 | 3.76 | 5.22 | 0.13 | **-** |
|  |  | #4 | 16.45 | 14.47 | 15.46 | 4.03 | 5.67 | 0.14 | **-** |
|  |  | #5 | 12.23 | 17.40 | 14.82 | 5.22 | 6.60 | 0.14 | **-** |
|  |  | #6 | 12.82 | 15.52 | 14.17 | 5.19 | 6.25 | 0.12 | **-** |
|  |  | **Mean** | **14.47** | **15.30** | **14.89** | **4.67** | **6.01** | **0.13** | **-** |
|  |  | **S.D.** | **1.71** | **1.26** | **0.42** | **0.62** | **0.51** | **0.01** | **-** |
| **Saline**  **+**  **Acu** | 1.0 Hz  1.0 mm | #1 | 16.20 | 15.19 | 15.70 | 4.52 | 13.45 | 0.80 | 12/16=0.75 |
|  |  | #2 | 16.21 | 14.87 | 15.54 | 5.83 | 12.36 | 0.67 | 29/43=0.67 |
|  |  | #3 | 13.26 | 14.50 | 13.88 | 4.06 | 12.27 | 0.84 | 9/11=0.82 |
|  |  | #4 | 15.40 | 14.00 | 14.70 | 3.99 | 11.76 | 0.73 | 12/17=0.71 |
|  |  | #5 | 17.83 | 12.67 | 15.25 | 5.22 | 11.93 | 0.67 | 10/13=0.77 |
|  |  | #6 | 17.98 | 14.70 | 16.34 | 5.36 | 12.99 | 0.69 | 13/18=0.72 |
|  |  | **Mean** | **16.15** | **14.32** | **15.23** | **4.83** | **12.46** | **0.73** | **0.74** |
|  |  | **S.D.** | **1.74** | **0.90** | **0.85** | **0.75** | **0.64** | **0.07** | **0.05** |
| **Saline** | - | #1 | 18.19 | 15.81 | 17.00 | 5.04 | 7.34 | 0.19 | 3/12=0.25 |
|  |  | #2 | 16.62 | 13.68 | 15.15 | 5.13 | 6.25 | 0.11 | 4/11=0.36 |
|  |  | #3 | 16.33 | 15.79 | 16.06 | 4.75 | 6.42 | 0.15 | 4/12=0.33 |
|  |  | #4 | 18.93 | 17.29 | 18.11 | 5.41 | 7.44 | 0.16 | 3/11=0.27 |
|  |  | #5 | 15.93 | 14.35 | 15.14 | 5.05 | 6.54 | 0.15 | 4/12=0.33 |
|  |  | #6 | 10.81 | 15.84 | 13.33 | 4.79 | 5.65 | 0.10 | 4/15=0.27 |
|  |  | **Mean** | **16.14** | **15.46** | **15.80** | **5.03** | **6.61** | **0.14** | **0.30** |
|  |  | **S.D.** | **2.85** | **1.27** | **1.66** | **0.24** | **0.68** | **0.03** | **0.05** |
